# Comparison of a time-resolved fluoroimmunoassay with a solid phase immunoradiometric assay the measurement of alphafetoprotein in amniotic fluid

**DOI:** 10.1155/S1463924687000282

**Published:** 1987

**Authors:** Edward J. Coombes, Brian J. Moody, Howard James, Ciaron Kelly

**Affiliations:** 1 Department of Chemical Pathology Salisbury General Infirmary Wiltshire Salisbury SP2 7SX UK; 2 Polytechnic of the South Bank London SE1 UK


Journal of Automatic Chemistry/*Journal of Clinical Laboratory Automation*, Vol. 9, No. 3 (July–September 1987), pp. 129–131

### Comparison of a time-resolved fluoroimmunoassay with a solid phase immunoradiometric assay for the measurement of alphafetoprotein in amniotic fluid

**Edward J. Coombes, Brian J. Moody, Howard James**
*Department of Chemical Pathology, Salisbury General Infirmary, Salisbury, Wiltshire SP2 7SX, UK*

**and Ciaron Kelly**
*Polytechnic of the South Bank, London SE1*

### Introduction

A new commercially available dissociation enhanced lanthanide fluoroimmunoassay (DELFIA) for the measurement of amniotic fluid α fetoprotein has been evaluated against a monoclonal antibody-based solid-phase immunoradiometric assay (IRMA) which has been the routine assay used by a Regional Amniotic Fluid Alphafetoprotein (AFP) Screening Laboratory for two years.

The DELFIA (LKB Wallac) AFP assay is a non-isotopic technique which utilizes a lanthanide metal (Europium) chelate label possessing highly fluorescent properties (Soini and Kojola [1]; Hemmila *et al.* [2]). The assay incorporates two monoclonal antibodies directed against two different immunogenic sites on the AFP molecule one of which is solid phase immobilized to the well of a microtitration strip.

The aim of this study was to assess the laboratory and clinical performance of this DELFIA.

### Materials and methods

For μ fetoprotein analyses, amniotic fluid μ fetoprotein (AFP) was estimated by two techniques.

*DELFIA AFP 1244-004 (LKB Wallac, PO Box 10, SF20101, Turku 10, Finland)*

This product is a second-generation DELFIA AFP kit. AFP standards (range 1–1000 KU/l), QC and unknown samples (25 μl) are added to individual wells of microtitration strips (12 wells per strip, eight strips per plate) containing immobilized monoclonal antibody. The microtitration wells act as both reaction chambers and as measurement cuvettes. 200 μl of a europium labelled second monoclonal antibody is pipetted into the wells. The strips of microtitration wells are shaken vigorously on an automatic shaking device (Varishaker, Dynatech) for 2 min then incubated for 1 h at room temperature with continuous gentle shaking. The liquid contents of the wells are aspirated and the wells washed six times with wash buffer (plate-washing step is also the separation step), using an automatic plate-washer (LKB 1296-022). 200 μl of enhancement solution (forms a new chelate with the europium ion, thus amplifying the fluorescence) is added to each well and the strips shaken gently for 5 min. The microtitration strips are allowed to stand for 15 min and then the fluorescence measured on an automated LKB Wallac 1230 Arcus fluorometer with automated data handling. A microtitration plate of 96 wells can be fully processed with full print-out of patient results in 10 min. AFP results were calculated in kU/l corrected for the sample dilution actor and expressed as MU/l. The shelf life is reported to be at least five months (manufacturer's information).

*IRMA (SUCROSEP*) AFP IRMA, Boots-Celltech Diagnostics Ltd, Slough, UK*

This immunoradiometric assay (IRMA) was performed using the protocol detailed by the manufacturer, α fetoprotein standard (range 1·23–712 kU/l) or unknown sample (50 μl) is incubated for 2 h at room temperature (15–30 °C) with 1251-labelled monoclonal antibody to α fetoprotein (100 μl) and solid phase anti α fetoprotein immunoglobulin (100 μl). During this incubation the assay tubes are shaken vigorously (300–350 $min^{-1}$) using an agitator (Sucroagitator, Boots-Celltech Diagnostics Ltd).

Following incubation the free and bound fractions are separated by the SUCROSEP sucrose layering non-centrifugation technique (Wright and Hunter [3]) using a semi-automatic instrument (Sucroseparator, Boots-Celltech Diagnostics Ltd). This allows assay tubes to be rapidly separated in 20-tube batches with a mean asssay separation time of approximately 50 min.

The radioactivity in the assay tubes was counted on a multihead gamma counter (LKB 1260 multigamma 11) over a 120 s period. α fetoprotein values for clinical specimens were calculated as kU/l by spline function. The values were then corrected for the sample dilution factor and expressed as MU/l α fetoprotein.

* *SUCROSEP is a trademark of Boots-Celltech Diagnostics Ltd.*

*Specimen analysis*

Before analysis each amniotic fluid was diluted with a serum pool of zero α fetoprotein free serum. Normally a 1 in 200 dilution was used, but occasionally a higher dilution of the amniotic fluid sample was necessary. The batches for the two techniques were commenced on the same day with each amniotic fluid assayed in duplicate.

*Amniotic fluid samples:* 174 aliquots of amniotic fluid were investigated. These were collected by transabdominal amniocentesis during the second trimester of pregnancy for a variety of clinical indications. These samples were centrifuged and the supernatant either used immediately or stored at −20 °C until analysis.

*Normal pregnancies:* 115 liquors from normal pregnancies were investigated. Ultrasound examination of these pregnancies showed no obvious fetal abnormality. The amniotic fluid AFP by IRMA was within normal laboratory reference limits and there was no evidence of the acetyl cholinesterase isoenzyme band on polyacrylamide gel electrophoresis (Chubb *et al.* [4]; Smith *et al.* [5]).

*Abnormal pregnancies:* 55 samples were classified as 'abnormal'. These pregnancies had been terminated and the fetuses confirmed to have a severe fetal abnormality (27 open spina bifida, 28 anencephaly).

*Equivocal pregnancies:* Four samples were also studied from 'equivocal pregnancies' for which the outcome of the pregnancy was a normal infant but the amniotic fluid α fetoprotein concentration by IRMA was significantly elevated.

*Table 1. Assay reproducibility of DELFIA and IRMA (intra- and inter-assay) based on repeated analyses of control pools. Prior to assays samples 1 and 2 were diluted 1/200, sample 3 was diluted 1/600.*

| Sample | Mean AFP concentration MU/l | | Intra-assay %CV ($N = 10$) | | Inter-assay %CV ($N = 10$) | |
|---|---|---|---|---|---|---|
| | DELFIA | IRMA | DELFIA | IRMA | DELFIA | IRMA |
| 1. | 26·1 | 27·5 | 1·98 | 1·84 | 3·72 | 3·33% |
| 2. | 75·9 | 86·6 | 1·70 | 2·72 | 6·09 | 5·69% |
| 3. | 163·1 | 183·9 | 6·04 | 3·30 | 7·60 | 4·92% |

*Statistical methods*

Regression analysis was performed using standard techniques.

### Results

*Assay reproducibility*

The intra- and inter-assay coefficients of variation (CV) for the DELFIA and IRMA were similar (table 1). The CV of the IRMA appeared to be better than the DELFIA at the highest AFP control pool concentration (sample 3).

*The relationship between amniotic fluid AFP levels measured by DELFIA and IRMA*

Regression analysis of results from 174 amniotic fluid samples (figure 1) falling in the range of AFP values from 3·4 to 584·0 MU/l analysed by both methods gave an excellent correlation coefficient of 0·997 (IRMA = 1·155 DELFIA + 0·946).

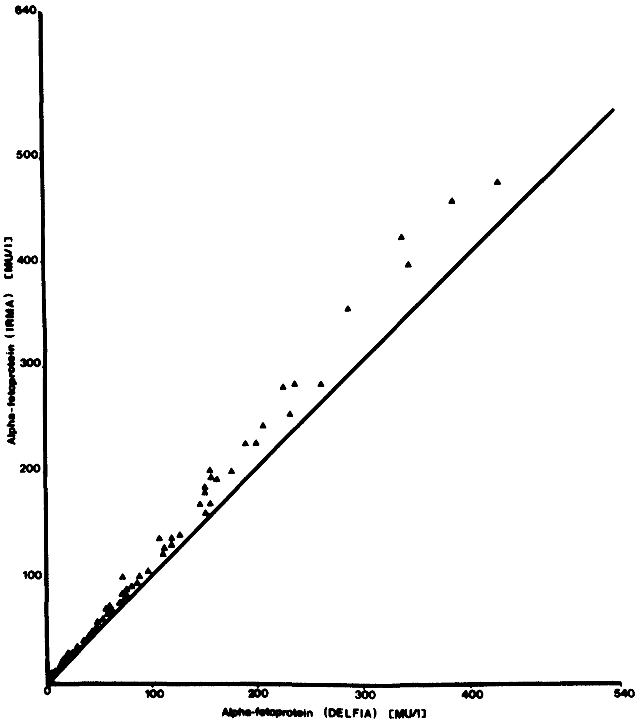

*Figure 1. Relationship between α fetoprotein values determined by DELFIA and IRMA for 174 amniotic fluid samples. Not all of the data points at the lower end of the graph are represented. The line indicated represents DELFIA = IRMA.*

*Table 2. α fetoprotein concentration of 115 amniotic fluids from normal pregnancies as measured by DELFIA.*

| Gestational age | Number of samples | Range of AFP values (MU/l) | Median AFP concentration | Recommended cutoff [6] level of AFP (MU/l) |
|---|---|---|---|---|
| 15 | 16 | 8·3–20·8 | 13·0 | 32·5 |
| 16 | 20 | 7·2–15·6 | 11·85 | 35·6 |
| 17 | 18 | 5·3–21·6 | 9·9 | 29·7 |
| 18 | 20 | 5·8–14·4 | 8·15 | 24·5 |
| 19 | 20 | 3·6–12·6 | 6·35 | 22·2 |
| 20 | 21 | 2·8–12·0 | 5·1 | 17·9 |

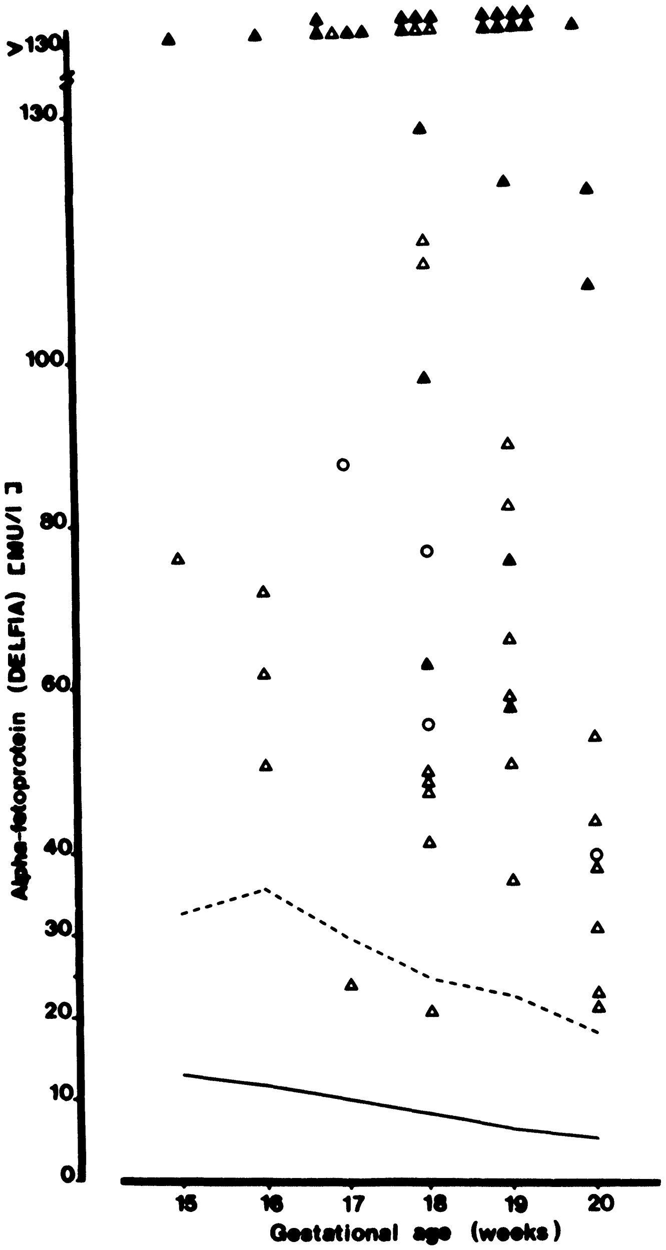

*Figure 2. α-fetoprotein values for 55 abnormal amniotic fluid samples measured by DELFIA. Where △ = spina bifida; ▲ = anencephaly; ○ = 'equivocal pregnancy'; —— median; - - - recommended multiple of the median cut-off level [6].*

*Clinical performance of DELFIA*

Table 2 indicates the median AFP concentration of the normal population for gestational ages of 15 to 20 weeks as measured by DELFIA.

The AFP levels of the 55 open neural tube defects and for comparison the median and multiple of the median cut off limits [6] of the normal pregnancy population are shown in figure 2.

In 53 of the 55 cases of open spina bifida and anencephaly the amniotic fluid AFP results as measured by DELFIA were greater than the multiple of the median cut-off limits. In two cases of open spina bifida the AFP level fell below the cut-off line (classified as false negative). The clinical classification of all 55 cases was exactly similar using either the DELFIA or the IRMA. In the remaining four amniotic fluids from the 'equivocal pregnancies' in which the outcome of the pregnancy was a normal infant, the AFP result as measured by DELFIA was elevated again, exactly similar to the results of the reference IRMA technique.

### Discussion

Excellent agreement was shown between the amniotic fluid AFP results ($r = 0{\cdot}997$) of the DELFIA and IRMA techniques. Furthermore the clinical performance of the two assays was exactly similar.

Both the DELFIA and IRMA are relatively quick techniques allowing a same day assay service if requried. The DELFIA assay was more rapid, largely due to the need for a 1 h incubation period compared to a 2 h period for the IRMA.

The equipment requirements of the two assays were similar, i.e. orbital shakers and a separating system (a plate-washer or the Sucroseparator), followed by a radioactivity counter or a fluorimeter. Both techniques do not require centrifugation steps.

The DELFIA offers a number of analytical advantages, a longer shelf life of reagents than conventional radioimmunoassays and IRMAs, a greater working range (up to 1000 kU/l), a smaller sample volume and a non isotopic technique which therefore dispenses with the complications surrounding the safe handling and disposal of radioactive material.

In summary, the DELFIA proved to be a sensitive, convenient, easy to use non isotopic alternative to the measurement of amniotic fluid α fetoprotein in the routine clinical chemistry laboratory.

### References

1. Soini, E. and Kojola, H., *Clinical Chemistry*, **29** (1983), 65.
2. Hemmila, I., Dakubu, S., Mukkala, V. M., Siitari, H. and Lorgren, T., *Annals of Biochemistry and Experimental Medicine*, **137** (1984), 335.
3. Wright, J. and Hunter, W. M., In: *Immonoassays for Clinical Chemistry*, edited by Hunter W. M. and Corrie, J. E. (Churchill Livingstone, Edinburgh, 1983).
4. Chubb, I. W., Pilowsky, P. M., Springwell, H. J. and Pollard, A. C., *Lancet*, **1** (1979), 688.
5. Smith, A. O., Wald, N. J., Cuckle, H. S., Stirrat, G. M., Bobrow, M. and Lagercrantz, H., *Lancet*, **1** (1979), 685.
6. Second report of the UK Collaborative Study on alpha-fetoprotein in relation to neural tube defects. *Lancet*, **11** (1979), 651.

